# Poor Weight Gain, Hypernatremia, and Jaundice in a 2-Month-Old Male

**DOI:** 10.1177/00099228221075412

**Published:** 2022-02-04

**Authors:** Thomas P. Swaffield, Sheila Clarke

**Affiliations:** 1 Penn State Health Children’s Hospital, Hershey, PA, USA

## Case Report

A 2-month-old male, born at 41 weeks via spontaneous vaginal delivery with a birth weight of 3990 g (75th percentile), presented to the emergency department (ED) with poor weight gain. His mother received routine prenatal care, but smoked during the pregnancy. His postnatal course was complicated by hyperbilirubinemia requiring phototherapy. At time of discharge from the nursery, his total bilirubin was below the threshold for phototherapy. His discharge weight was 3796 g down 4.8% from birthweight.

At his 2-week follow-up visit, he remained below birth weight. His newborn screen was normal, but he was admitted to an outside hospital for dehydration and poor weight gain. Laboratory results from workup of poor weight gain included hypernatremia of 152 mEq/L (152 mmol/L; normal range = 135-145 mEq/L). During that admission, his formula was changed from 19 calories/ounce to 22 calories/ounce with resultant appropriate weight gain. Discharge weight was not available from his hospital discharge summary, but his sodium level remained elevated at 149 mEq/L (149 mmol/L; normal range = 135-145 mEq/L). Available records did not indicate any follow-up to address the persistent hypernatremia. His weight was recorded as 4252 g at his 1-month follow-up visit, indicating suboptimal weight gain of 9 g/day since birth. His parents were advised to present to the nearest ED for further workup.

In the ED, his mother reported his feeds as approximately 120 mL of 22 calories/ounce formula every 2 to 3 hours, with a description of appropriate formula preparation. In addition, she reported occasional emesis and loose stools, which improved after changing to a soy-based formula in the previous week. Stools at that time were described as non-bloody and watery. She also reported that he had approximately 10 wet diapers per day, but had no other concerns.

Admission examination revealed an afebrile infant with other vital signs within normal limits for age, weight of 4555 g (10th percentile), head circumference 38 cm (23rd percentile), and length 56 cm (20th percentile). He appeared malnourished, jaundiced, and had bilateral eye discharge without conjunctival injection. Abdominal examination was benign, without any palpable masses or hepatosplenomegaly. Genitourinary examination revealed normal external male genitalia and bilateral descended testes. The rest of his examination was unremarkable.

**Figure 1.**
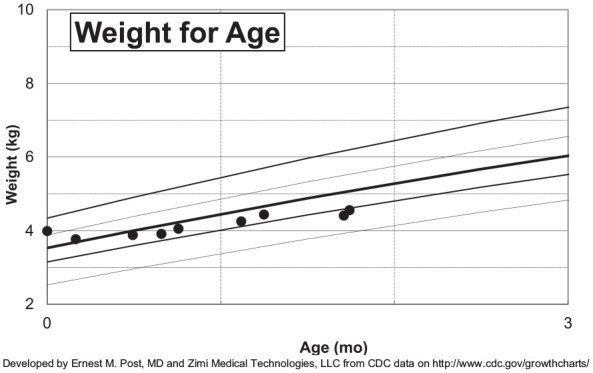
Infant’s growth chart from birth to 2 months of age. Weights shown are based on history from mother and chart review, generated with E.M. Post and Zimi Medical Technologies Excel Based Clinical Tools to Assist with Growth Charts.^13^

Laboratory values were significant for hypernatremia 152 mEq/L (152 mmol/L; normal range = 135-145), mild hyperkalemia 5.5 mEq/L (5.5 mmol/L; normal range = 3.5-5.3), hyperchloremia 112 mEq/L (112 mmol/L; normal range = 98-107), and hyperbilirubinemia, with total bilirubin and direct bilirubin measuring 3.9 mg/dL (66.6 µmol/L; normal range = 0.0-20.5) and 2.4 mg/dL (41.0 µmol/L; normal range = 0.0-5.1), respectively. Bicarbonate was normal at 26 mmol/L (normal range = 22-29), blood urea nitrogen and creatinine were 17 mg/dL (6.1 mmol/L; normal range = 2.1-8.2) and 0.31 mg/dL (27.4 mmol/L; normal range = 17.7-35.4), respectively. Liver panel showed elevated alkaline phosphatase of 627 U/L (normal range = 122-469), aspartate aminotransferase of 55 U/L (normal range = 0-40), and γ-glutamyl transferase of 66 U/L (normal range = 8-61). Urine specific gravity was 1.009 (normal range = 1.005-1.030) and remainder of urinalysis was unremarkable. Urine osmolality and serum osmolality were 157 mOsm/kg (157 mmol/kg; normal range = 100-1000) and 320 mOsm/kg (320 mmol/kg; normal range = 275-295), respectively. Complete blood count was normal. The patient was admitted for further evaluation of poor weight gain (Figure 1) in the setting of multiple abnormal laboratory results.

## Final Diagnosis

The initial differential diagnoses list included biliary atresia, malabsorption, and diabetes insipidus (DI). A final diagnosis of septo-optic dysplasia (SOD) with associated optic nerve hypoplasia, pituitary hypoplasia with adrenal insufficiency, and growth hormone deficiency, as well as other midline brain abnormalities was made.

## Hospital Course

**Figure 2.**
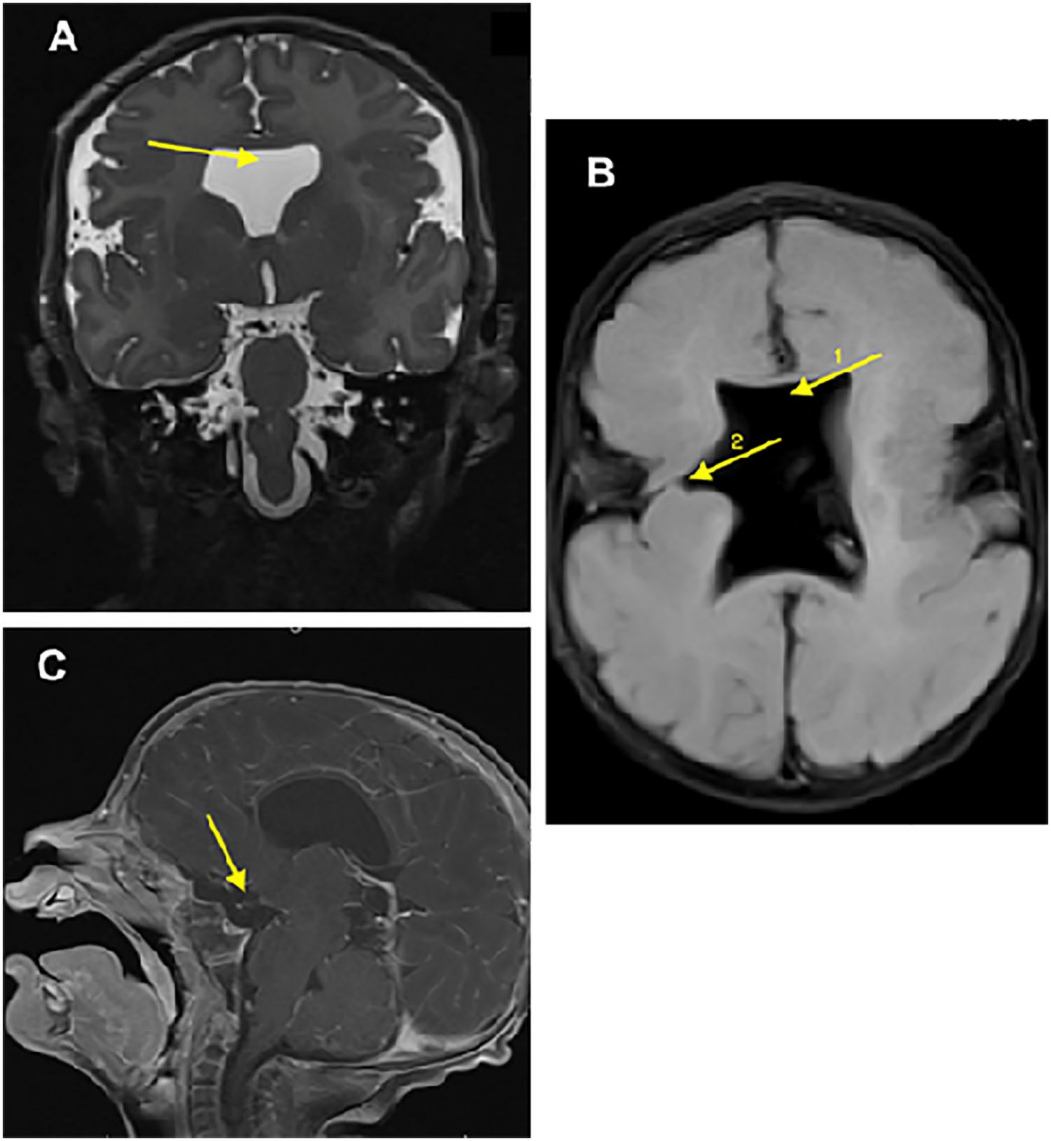
T2-weighted brain magnetic resonance imaging (MRI) images showing an absent septum pellucidum in the coronal (A) and axial (B) views (arrow 1), as well as schizencephaly (arrow 2) in the axial view. T1-weighted brain MRI image in sagittal view showing midline structures, hypoplastic pituitary gland, and optic chiasm (C).

On admission, workup was initiated for poor weight gain, hypernatremia, and conjugated hyperbilirubinemia. DI was suspected due to persistent hypernatremia, elevated serum osmolality, and low urine specific gravity and osmolality. A trial of desmopressin confirmed central rather than nephrogenic DI. His presentation with DI, conjugated hyperbilirubinemia, and poor weight gain raised the suspicion for the possibility of panhypopituitarism, since central DI is rarely an isolated defect. A brain magnetic resonance imaging (MRI) done to evaluate the pituitary stalk revealed findings consistent with SOD including optic nerve and pituitary hypoplasia, absent septum pellucidum, and other midline brain abnormalities (Figure 2).

Further studies performed to evaluate pituitary function showed growth hormone deficiency and normal thyroid function. ACTH (adrenocorticotropic hormone) stimulation testing showed inadequate cortisol production consistent with secondary adrenal insufficiency. He was subsequently started on hydrocortisone and growth hormone replacement. An abdominal ultrasound done to exclude biliary atresia as part of the evaluation for conjugated hyperbilirubinemia was normal. The diagnosis of central DI, panhypopituitarism, and a normal abdominal ultrasound explained the conjugated hyperbilirubinemia, thus further workup to exclude biliary atresia was not pursued. Resolution of loose stools in the setting of normal albumin, negative stool tests for malabsorption and infectious causes, excluded gastrointestinal etiology for his poor weight gain.

Renal ultrasound and echocardiogram obtained to exclude other midline defects were normal. Conjugated hyperbilirubinemia improved with treatment of his endocrine deficiencies, and he had adequate weight gain on a low sodium formula. Eye discharge was due to dacryostenosis and resolved with appropriate treatment. He was discharged on desmopressin, growth hormone, and hydrocortisone with Endocrine, Ophthalmology, Neurology, and Genetics specialty follow-up. All the specialty services were consulted during his admission.

## Discussion

Septo-optic dysplasia is a heterogeneous, difficult, and rare diagnosis, equally prevalent in males and females, occurring in 1 in 10 000 infants.^1^ Two or more features of the classic triad, optic nerve hypoplasia, pituitary hormone abnormalities, and midline brain defects including agenesis of the septum pellucidum and/or corpus callosum, are required for diagnosis^1^ (Figure 2). It is debatable whether milder variants occur, as isolated features do not qualify for the diagnosis of SOD.^1^ About 30% of SOD cases have complete manifestations, 62% display hypopituitarism, and 60% have absent septum pellucidum.^1^

Septo-optic dysplasia results from an abnormality of early forebrain development occurring in the first trimester, usually associated with pituitary dysfunction. The exact etiology is unknown, but most cases are sporadic. It is likely multifactorial with both environmental and genetic factors.^2^ Genetic mutations in at least 4 developmental genes, which include heterozygous mutations in HESX1, SOX2, SOX3, and OXT3 genes, is seen in <1% of patients.^3^ There is also a suggestion that SOD is the result of vascular disruption,^4^ as well as other miscellaneous factors including young maternal age, drug, and alcohol use.^5^

The main clinical findings in SOD are hypopituitarism (62% to 80%), with growth hormone deficiency being the most commonly affected hormone, visual impairment (23% have significant impairment), and developmental delay. The most frequent neurological manifestations are seizures, developmental delay, and cerebral palsy.^1^ Most children present within the first 2 years of life, but infants with subtle presentations can be diagnosed earlier with astute clinical suspicion. Findings that should raise suspicion for SOD include hypoglycemia, conjugated hyperbilirubinemia, micro phallus with or without cryptorchidism, and nystagmus. Midline abnormalities such as cleft palate may also be present.^1^

A brain MRI in conjunction with assessment of pituitary function and ophthalmological evaluation should be used to confirm the diagnosis of SOD.^1,6^ Pituitary function tests include obtaining thyroid-stimulating hormone and free T4, growth hormone, and cortisol level. If random cortisol level is low, stimulation testing should be performed.^1^ The MRI on almost all children with central DI shows brain abnormalities, the most common being central nervous system (CNS) malformations and intracranial masses.^7^ Other studies corroborate the preponderance of CNS abnormalities and idiopathic etiology is rare.^8^

Inadequate intake is the most common reason for poor weight gain. Up to 90% of poor or inadequate weight gain will have no underlying medical reason apart from insufficient calorie intake on further workup.^9^ However, insufficient calories did not explain our patient’s presentation since his mother demonstrated appropriate formula preparation, and his caloric intake was adequate for his age. Further workup was indicated to evaluate his poor weight gain. Hypernatremia is most commonly associated with dehydration; however, when signs of clinical dehydration are present in the context of adequate intake, disruption of antidiuretic hormone should be considered.^10^ DI was suspected due to persistent hypernatremia, elevated serum osmolality, low urine osmolality, and specific gravity in the setting of increased urine output. In addition, our patient presented with conjugated hyperbilirubinemia, which should always be investigated for underlying pathology.^9^ Biliary atresia is one of the most common causes of neonatal conjugated hyperbilirubinemia, and timely diagnosis with intervention are necessary to ensure an optimal outcome. Other important causes to consider include genetic, endocrine, and metabolic abnormalities.^9^ Once the diagnosis of central DI was established, brain imaging was indicated to assess for further abnormalities, as idiopathic central DI is rare.

Management of SOD involves a multidisciplinary approach including Endocrine, Neurology, Ophthalmology, Early Intervention, and Genetics consultation. Close monitoring from these specialties ensures that appropriate interventions can be implemented in a timely manner. Our patient was started on daily desmopressin injections, a low sodium formula, as well as growth hormone and hydrocortisone replacements. Some studies suggest treatment with thiazide diuretic as another option in this age group and may be ideal given the ease of administration in oral form. However, no studies show a difference in effectiveness comparing desmopressin to thiazides.^6^ Parents received extensive education on medication administration prior to discharge, including administering steroid stress doses for periods of illness, which is an important aspect to the long-term care of these patients.

## Conclusion

Poor weight gain is usually due to inadequate caloric intake. It is easily excluded with monitoring weight gain on adequate calories. The differential diagnosis is broad and can be narrowed by a thorough history, physical examination, and guided laboratory testing. Abnormal laboratory results should be further investigated.^9^ DI should always be suspected and evaluated in patients presenting with appropriate formula preparation, with or without dehydration, and persistent hypernatremia associated with increased dilute urine output.^10^ Isolated or idiopathic central DI is rare; therefore, further evaluation for panhypopituitarism and CNS abnormalities are mandatory.^8^

Both indirect and direct hyperbilirubinemia are associated with hypopituitarism, and resolve with replacement of growth hormone and hydrocortisone.^11^ Cholestatic jaundice is never benign and should be evaluated, especially to exclude biliary atresia as surgical intervention prior to 2 months improves the outcome.^12^ It is imperative that these patients be closely followed by specialists in addition to their primary care pediatricians.

## Author Contributions

TPS: Contributed to conception; contributed to literature review; drafted the manuscript; agrees to be accountable for all aspects of work ensuring integrity and accuracy.

SC: Contributed to conception; contributed to literature review; critically revised the manuscript; gave final approval; agrees to be accountable for all aspects of work ensuring integrity and accuracy.
